# Molecular investigation of endoparasites of marine mammals (Cetacea: Mysticeti, Odontoceti) in the Western Mediterranean

**DOI:** 10.3389/fvets.2024.1431625

**Published:** 2024-09-10

**Authors:** Nicolas R. Specht, Gergő Keve, Carolina Fernández-Maldonado, Alejandra Cerezo Caro, Nóra Takács, Jenő Kontschán, Sándor Hornok

**Affiliations:** ^1^Department of Parasitology and Zoology, University of Veterinary Medicine, Budapest, Hungary; ^2^HUN-REN-UVMB Climate Change: New Blood-Sucking Parasites and Vector-Borne Pathogens Research Group, Budapest, Hungary; ^3^Seashore Environment and Fauna, Tarifa, Spain; ^4^Plant Protection Institute, Centre for Agricultural Research, Budapest, Hungary; ^5^Department of Plant Sciences, Albert Kázmér Faculty of Mosonmagyaróvár, Széchenyi István University, Mosonmagyaróvár, Hungary

**Keywords:** Brachycladiidae, Cestoda, Nematoda, Phyllobothriidae, Pseudaliidae, *Toxoplasma gondii*, Trematoda, cetacea

## Abstract

**Introduction:**

Whales, dolphins, and porpoises are susceptible to infections by protozoan and metazoan parasites.

**Methods:**

In this study, tissue samples, as well as flatworms and roundworms, were collected from a common bottlenose dolphin (*Tursiops truncatus*), three short-beaked common dolphins (*Delphinus delphis*), two striped dolphins (*Stenella coeruleoalba*), a harbor porpoise (*Phocoena phocoena*), a long-finned pilot whale (*Globicephala melas*), and a fin whale (*Balaenoptera physalus*). These samples were molecularly analyzed.

**Results:**

In one *D. delphis, Toxoplasma gondii* was detected in multiple organs, including the cerebellum. The cysts of the tapeworms *Clistobothrium delphini* and *Clistobothrium grimaldii* were identified in *G. melas*. Flukes collected from *D. delphis* belong to *Brachycladium atlanticum*, while those removed from *S. coeruleoalba* probably represent a new species. Four species of lungworms were also identified: *Halocercus delphini* in *S. coeruleoalba*, *Halocercus* sp. in *T. truncatus*, *Stenurus globicephalae* in *G. melas*, and a potentially new *Pharurus* sp. in *P. phocoena*.

**Conclusion:**

These findings show, to the best of our knowledge, for the first time, the presence of *T. gondii* DNA in *D. delphis*. The cerebellum of the animal was *Toxoplasma*-infected, which might be relevant to inadvertent stranding. In this study, new genetic markers were sequenced for several helminth parasites of marine mammals, possibly including undescribed species.

## Introduction

Cetaceans (Artiodactyla: Cetacea), including whales, dolphins, and porpoises, are streamlined aquatic mammals that spend their whole life in water. All of them are carnivorous, taking either many small preys by bulk filter-feeding (parvorder Mysticeti: baleen whales) or larger prey by echolocation-assisted hunting (parvorder Odontoceti: dolphins, porpoises, toothed whales) (1). Cetaceans include the largest animals of all extinct and extant species and range through all oceans and into some rivers (1).

Cetaceans living under sea conditions represent an integral part of marine ecosystems. Many of their representatives are highly protected, or even critically endangered species. This makes it especially important to study pathogenic microorganisms (viruses, bacteria, and protozoa) as well as parasitic worms they might carry because some of these severely impact their health status (2). This is especially true in an era of climate change when seawater is becoming increasingly contaminated with land-derived microorganisms, such as waterborne bacteria and parasites, that reach the marine environment from freshwaters on the continental mainland due to flooding (3, 4). The most relevant examples of marine or land-derived pathogens affecting cetaceans are those that may underlie their frequently enigmatic stranding, a phenomenon that represents a global problem but also affects the ecosystems of the Mediterranean Sea (5).

At the same time, cetaceans also play a significant role in the epidemiology of those pathogens that have high veterinary-medical significance from the point of view of a broader range of mammals (6), even human beings. The latter is well-exemplified by *Toxoplasma gondii* (7). Although in most countries with maritime boundaries, marine mammals are usually not caught or directly used for human consumption, they can still participate in the natural transmission cycle of zoonotic bacteria (8) and protozoan parasites (9).

Cetaceans of the Mediterranean Sea include at least six endangered species, and two others categorized as vulnerable (10). Until the era of molecular methods, studies on their pathogenic microbiota were frequently based on serological tests, which are also used nowadays (11). However, detecting systemic antibodies is not always informative on the active status of infection and frequently precludes the organ-specific evaluation of potential causes. Another shortcoming of available data is relevant to groups of protozoan parasites in the marine environment that may cause neurological diseases [e.g., *Acanthamoeba*: (12)], but for which marine mammals are seldom if ever tested. Last but not least, molecular studies on the helminth fauna of cetaceans appear to show an increasing tendency, with potentially new species also discovered in the marine environment surrounding continental Europe [e.g., (13)]. Despite this, only a minority of worm species associated with cetaceans were barcoded, and only some of them have broadly accessible sequence data in GenBank (e.g., among lung-associated nematodes, only 4 out of the 13 *Halocercus* spp.).

The present study was initiated to contribute to our knowledge in the above context. Tissue samples were obtained from various organs of nine individuals of six cetacean species that were found dead in the coastal area of the Strait of Gibraltar (southern Spain). These samples were examined with polymerase chain reaction (PCR) and sequencing for the presence of protozoan parasites known to cause parasitemia and neurologic diseases in marine and/or other mammals, such as *Acanthamoeba* and cystogenic coccidia. In addition, the worms recovered during pathological examination, representing all three major groups (flukes, cestodes, and nematodes), were evaluated taxonomically with molecular-phylogenetic analyses.

## Materials and methods

### Sample collection

Tissue and parasite samples were collected from stranded and dead cetaceans [a common bottlenose dolphin (*Tursiops truncatus*), three short-beaked common dolphins (*Delphinus delphis*), two striped dolphins (*Stenella coeruleoalba*), a harbor porpoise (*Phocoena phocoena*), a long-finned pilot whale (*Globicephala melas*), and a fin whale (*Balaenoptera physalus*)] during official necropsies regularly conducted in Algeciras, Spain, between August and December 2022. The necropsies were led by the veterinary team of the NGO Seashore Ambiental at the facilities of the Center for Management of Marine Environment (CEGMA), which belongs to Clearwater Marine Aquarium Sea (CMASEA), Andalusian Regional Government (AMAYA), and is responsible for managing and coordinating the stranding network in Andalusia through its Marine Environment Program.

A sampling protocol and pre-numbered tube sampling system were implemented in the fieldwork. All relevant data were recorded according to tube numbers, including place and date of finding, the name of cetacean species, and any observable information regarding age, sex, and general condition of the individual. The organ location of parasites and the parasite load were also documented, and pictures of lesions and parasites were made on the premises.

Tissue samples were taken from the muscle, lungs, liver, spleen, blood, medulla, cerebellum, and encephalon.

Nematodes and flukes were placed into 96% pure ethanol. Because of varying degrees of autolysis, their morphological identification was not attempted. Native tissue samples (removed with sterile scalpel blades or scissors from the middle of the relevant organ) were placed into empty 2 mL or 9 mL prelabelled screw-cap Sarstedt tubes (Sarstedt, Nümbrecht, Germany), depending on the size of the sample. The tissue samples were frozen at −20°C until use, but the worms soaked in ethanol were stored at room temperature. In this way, 31 tissue and 17 parasite samples were collected from 9 cetacean individuals (Supplementary Table S1).

### DNA extraction and molecular analyses

DNA was extracted using the QIAamp DNA Mini Kit (QIAGEN, Hilden, Germany), following the manufacturer’s instructions. Extraction controls (tissue lysis buffer) were processed with each set of samples to monitor cross-contamination.

PCR reactions are summarized according to primers and cycling conditions in Supplementary Table S2. The reaction components were included in a volume of 25 μL, containing 1 U (stock 5 U/μl) HotStarTaq Plus DNA Polymerase, 2.5 μL of 10 × CoralLoad reaction buffer (including 15 mM MgCl_2_), 0.5 μL of PCR nucleotide Mix (stock 10 mM), 0.5 μL of each primer (stock 50 μM), 15.8 μL of ddH_2_O, and 5 μL of template DNA. In all PCRs, a non-template reaction mixture served as the negative control. The extraction controls and negative controls remained PCR-negative in all tests.

### Sequencing and phylogenetic analyses

Purification and sequencing of the PCR products were performed by Biomi Ltd. (Gödöllő, Hungary). Quality control and trimming of sequences were performed with the BioEdit program. Obtained sequences were compared to GenBank sequences by the nucleotide BLASTN program.^1^ Sequences were submitted to GenBank (Table 1).

**Table 1:** Parasite genera and species are shown according to the target gene and GenBank accession numbers of sequences based on which they were identified in this study.

Parvorder: family	Host species	Parasite group (genus and/or species)	GenBank accession numbers according to target gene		
			Large (small) subunit: *28S (18S) rRNA*	Internal transcribed spacer: ITS2	Cytochrome *c* oxidase subunit I: *Cox1*
**Odontoceti**: Delphinidae	Short-beaked common dolphin (*Delphinus delphis*)	*Toxoplasma gondii*	(OR881389)	–	–
		Flukes (*Brachycladium atlanticum*)	–	OR886409	OR881384
	Striped dolphin (*Stenella coeruleoalba*)	Lungworms (*Halocercus delphini*)	–	OR900631	OR885546, OR885547
		Flukes (*Brachycladium* sp. Cádiz)	–	OR886408	OR881381, OR881382, OR881383
	Common bottlenose dolphin (*Tursiops truncatus*)	Lungworms (*Halo*ce*rcus* sp. Cádiz)	–	–	OR885545, OR885548, OR885549
	Long-finned pilot whale (*Globicephala melas*)	Lungworms (*Stenurus globicephalae*)	–	OR900632	OR88550, OR88551
		Cestodes (*Clistobothrium delphini*)	OR886384	OR886386	OR885543, OR885544
		Cestodes (*Clistobothrium grimaldii*)	OR886385	OR886387	–
**Odontoceti**: Phocoenidae	Harbor porpoise (*Phoconea phoconea*)	Lungworms (*Pharurus* sp. Cádiz)	–	–	OR885552, OR885552

Sequences from other studies (retrieved from GenBank) included in the phylogenetic analyses had approximately or exactly 100% coverage with sequences from this study. Sequence datasets were resampled 1,000 times to generate bootstrap values. Phylogenetic analyses were conducted with the maximum likelihood method with the MEGA version 7.0 software.

## Results

### Molecular screening and analyses of protozoan parasites

All tissue DNA extracts were negative for *Acanthamoeba* spp. However, in the general PCR for cystogenic coccidia (Apicomplexa: Sarcocystidae), four samples were positive. These originated from the lungs, muscles, cerebrum, and cerebellum of the same short-beaked common dolphin. In these organs, *Toxoplasma gondii* was detected with sequencing (Table 1). The amplified part of the small-subunit *18S rRNA* gene showed 100% identity with several GenBank sequences, including the highly pathogenic RH strain (e.g., EF472967 and U17349). This was confirmed with *Toxoplasma*-specific primers of the repeat region in its genome. The liver, spleen, and blood samples of the same animal were PCR-negative.

### Molecular analyses of tapeworms (Cestoda: Phyllobothriidae)

**Figure 1:**
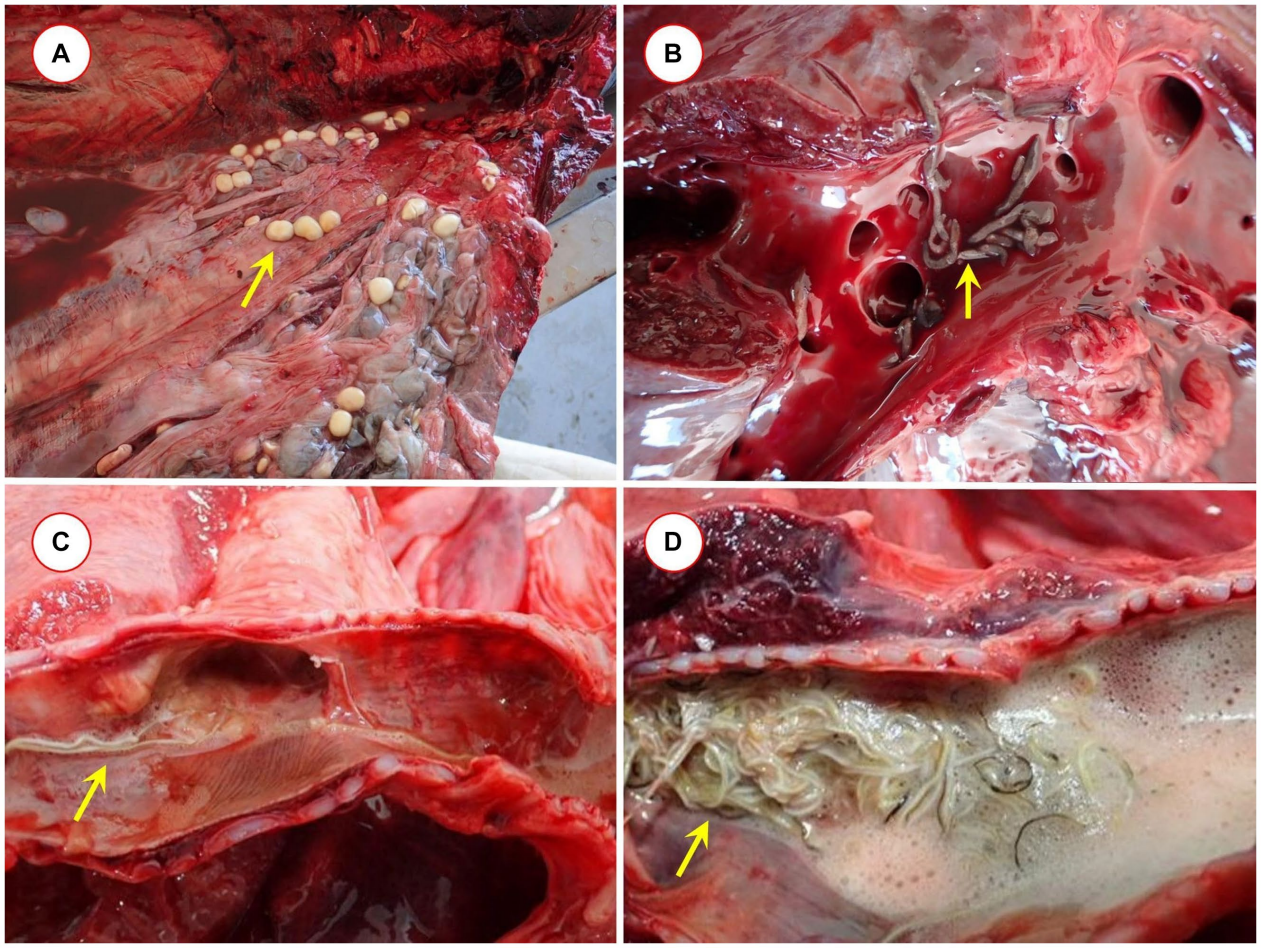
Pathological findings: **(A)** *Clistobothrium* sp. in the blubber of *Globicephala melas*, **(B)** *Brachycladium* sp. in the hepatic ducts of *Stenella coeruleoalba*, and **(C)** and **(D)** *Halocercus* sp. in the respiratory passages of *Tursiops truncatus*.

Cysts (merocercoids) of cestodes were present in the blubber of a long-finned pilot whale (Figure 1A). The ITS2 sequences of the two larvae were only 97.8% identical to each other, suggesting that they belonged to different species. The longer part of the *cox1* gene was also successfully amplified from one of the cysts, and its ITS2 sequence confirmed its closest relationship with the genus *Clistobothrium*, with 98.6% ITS2 and 89.6% *cox1* sequence identity to previously reported sequences (KU724058 and KU987913, respectively) from cape fur seals (*Arctocephalus pusillus*). Another, shorter (377 bp) part of the *cox1* gene showed clustering of this isolate as a sister group to members of the family Phyllobothriidae, including the genus *Clistobothrium*, although with low support (Supplementary Figure S1).

**Figure 2:**
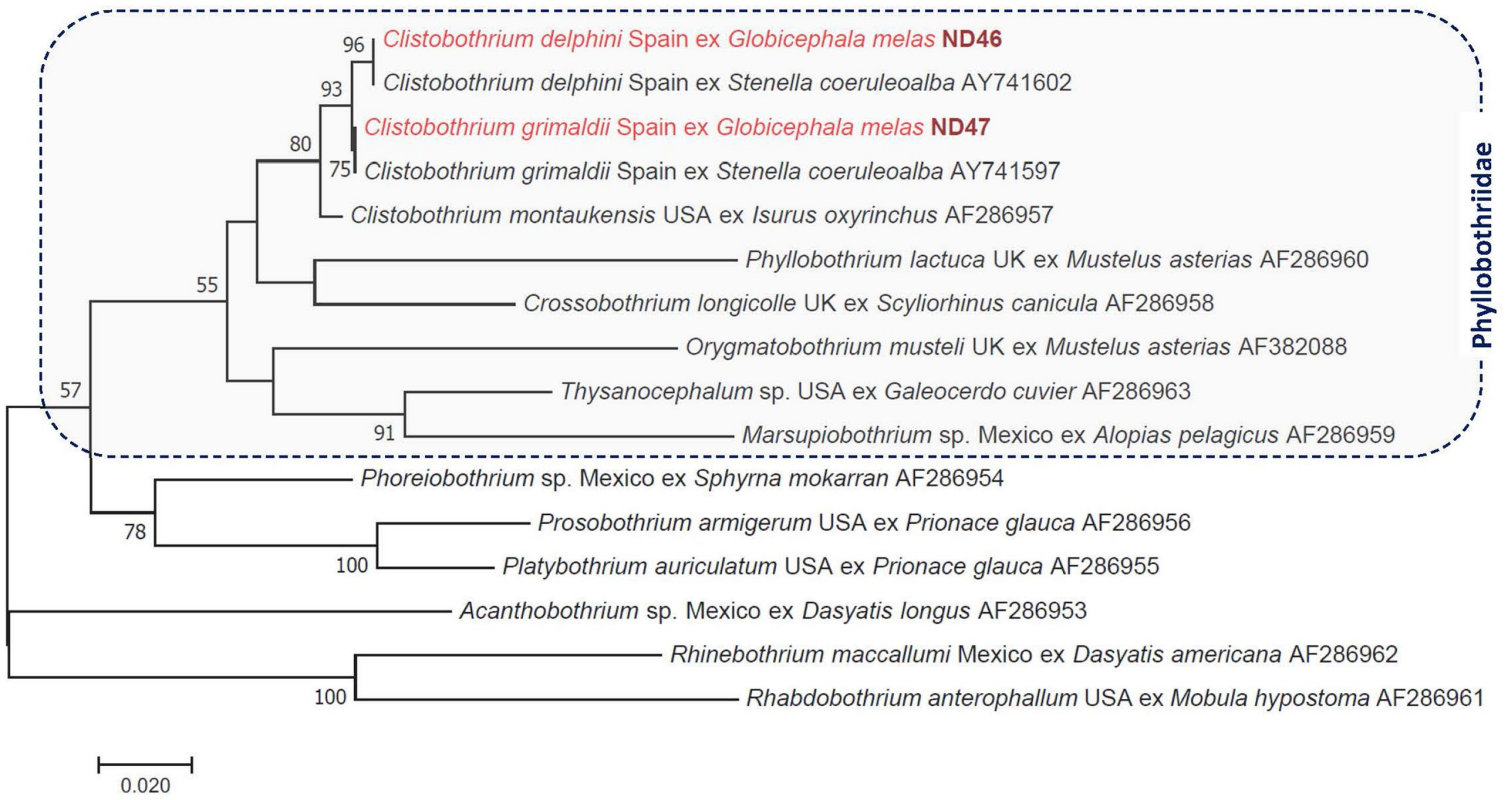
Phylogenetic tree of tapeworms (Cestoda: Phyllobothriidae, Onchobothriidae, Rhinebothriidae) based on the *28S rRNA* gene. In each row of sequences, after the helminth species name, the isolation source (host), the country or region of origin, and the GenBank accession number are shown. Sequences from this study are indicated with red fonts and bold, maroon accession numbers. The evolutionary history was inferred by using the maximum likelihood method based on the general time-reversible model. The tree is drawn to scale, with branch lengths measured in the number of substitutions per site. The analysis involved 16 nucleotide sequences. All positions containing gaps and missing data were eliminated. There were a total of 639 positions in the final dataset.

A 1280 bp-long fragment of the *28S rRNA* gene was also successfully amplified from both cysts. One of them, the same as above, showed 100% sequence identity to *Clistobothrium delphini* from a striped dolphin reported previously from Spain (AY741600). The other had 100% sequence identity to *Clistobothrium grimaldii* from *A. pusillus* sampled in Japan (LC718556). Based on the *28S rRNA* gene, all three *Clistobothrium* species included in the phylogenetic analysis formed a monophyletic clade, in which the clustering of *C. delphini* and *C. grimaldii* was well-supported (with 93%: Figure 2). The ITS2 or *cox*1 sequences of these tapeworm species were not available in GenBank for comparison.

### Molecular analyses of flukes (Trematoda: Brachycladiidae)

Flukes were collected from two species of dolphins (Figure 1B). The fluke found on the peritoneum and in the liver of a short-beaked common dolphin was molecularly identified as *Brachycladium atlanticum*, on account of its 100% ITS2 sequence identity to this species (FJ211250). The *cox1* sequence of *B. atlanticum* was 89.5% identical to that of *B. goliath* (NC_029757) and was hitherto not available, but was submitted from this study to GenBank.

**Figure 3:**
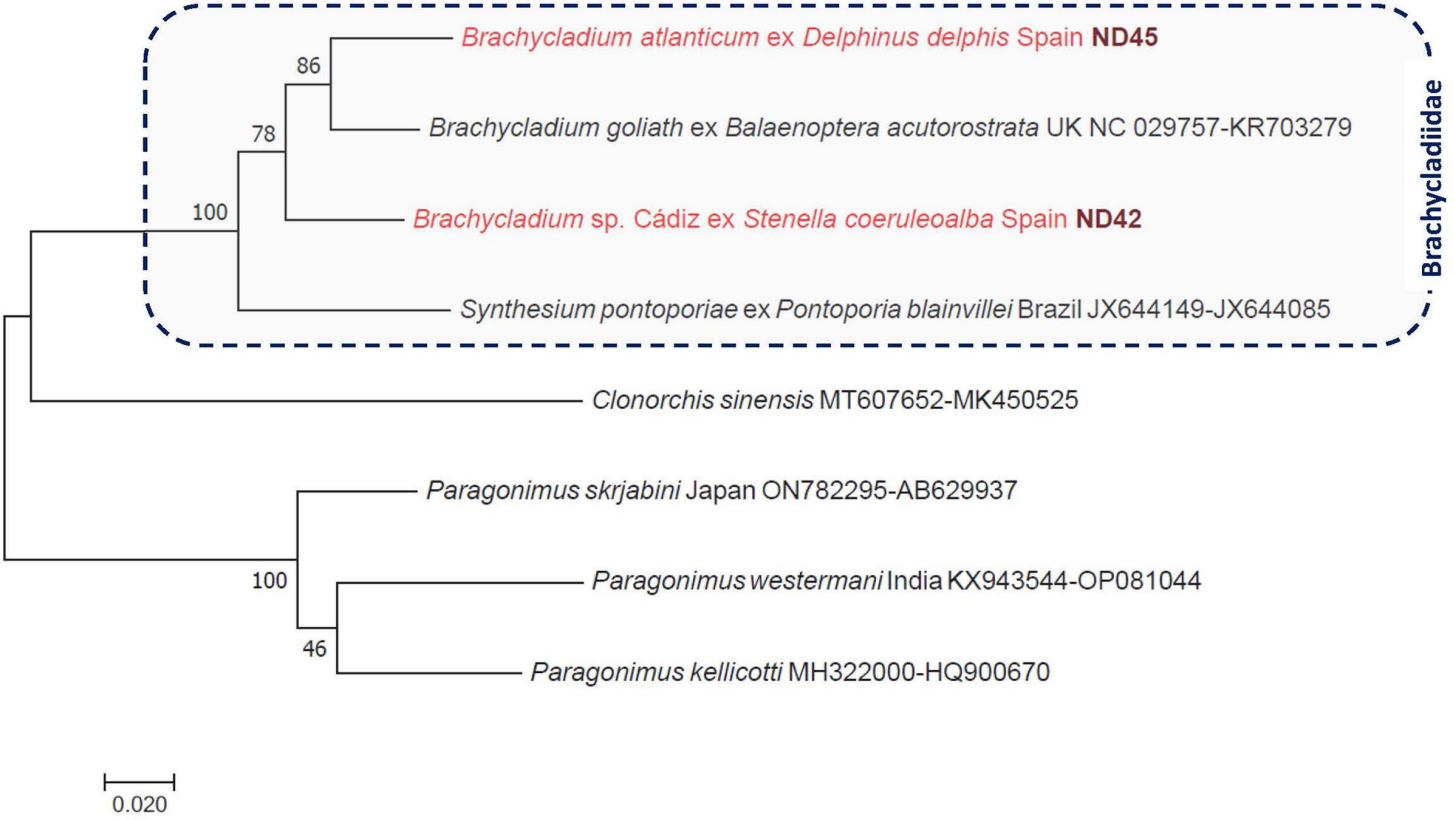
Phylogenetic tree of digenetic flukes (Trematoda: Brachycladiidae, Opisthorchiidae, Paragonimidae) based on concatenated *cox1* and ITS2 sequences. In each row of sequences, after the helminth species name, the isolation source (host), the country or region of origin, and the GenBank accession number are shown. Sequences from this study are indicated with red fonts and bold, maroon accession numbers. The evolutionary history was inferred by using the maximum likelihood method based on the general time-reversible model. The tree is drawn to scale, with branch lengths measured in the number of substitutions per site. The analysis involved eight nucleotide sequences. All positions containing gaps and missing data were eliminated. There were a total of 811 positions in the final dataset.

In the case of flukes from the pylorus, gallbladder, and hepatic ducts of a striped dolphin (*n* = 3), the *cox1* gene sequences indicated slight intraspecific genetic variation and only 87–87.5% sequence identity to the most closely related species, *Brachycladium goliath* (NC_029757). The ITS2 sequences of these worms were identical to each other and most similar to that of *B. goliath*, meaning 98% identity to the corresponding sequence (KR703279) of this species. The phylogenetic analyses of concatenated genes confirmed these results, and the species from *S. coeruleoalba* (provisionally called here *Brachycladium* sp. Cádiz) clustered separately from both *B. goliath* and *B. atlanticum*, with moderately high (78%) support (Figure 3).

### Molecular analyses of lungworms (Nematoda: Pseudaliidae)

Lungworms were found in the respiratory tracts of four cetacean species (Figures 1C,D): *S. coeruleoalba*, *T. truncatus*, *G. melas*, and *P. phocoena*. In *S. coeruleoalba*, two genetic variants of *Halocercus delphini* were identified, according to their 96.6–99.2% *cox1* sequence identity to a formerly reported sequence of this species (OQ200459). In *T. truncatus*, a different species (provisionally named *Halocercus* sp. Cádiz) showed only 93.9–94.4% *cox1* sequence identity to the most closely related species, *Halocercus pingi* (OQ200469). From the pterygoid sinus of *G. melas*, *Stenurus globicephalae* was identified, based on its 99.1–99.4% *cox1* sequence identity to this species (OQ200456), and in *P. phocoena*, a *Pharurus* species (tentatively called *Pharurus* sp. Cádiz), which showed the highest but only 90.2–90.6% *cox1* sequence identity to *Phanurus sunameri* (OQ200467).

**Figure 4:**
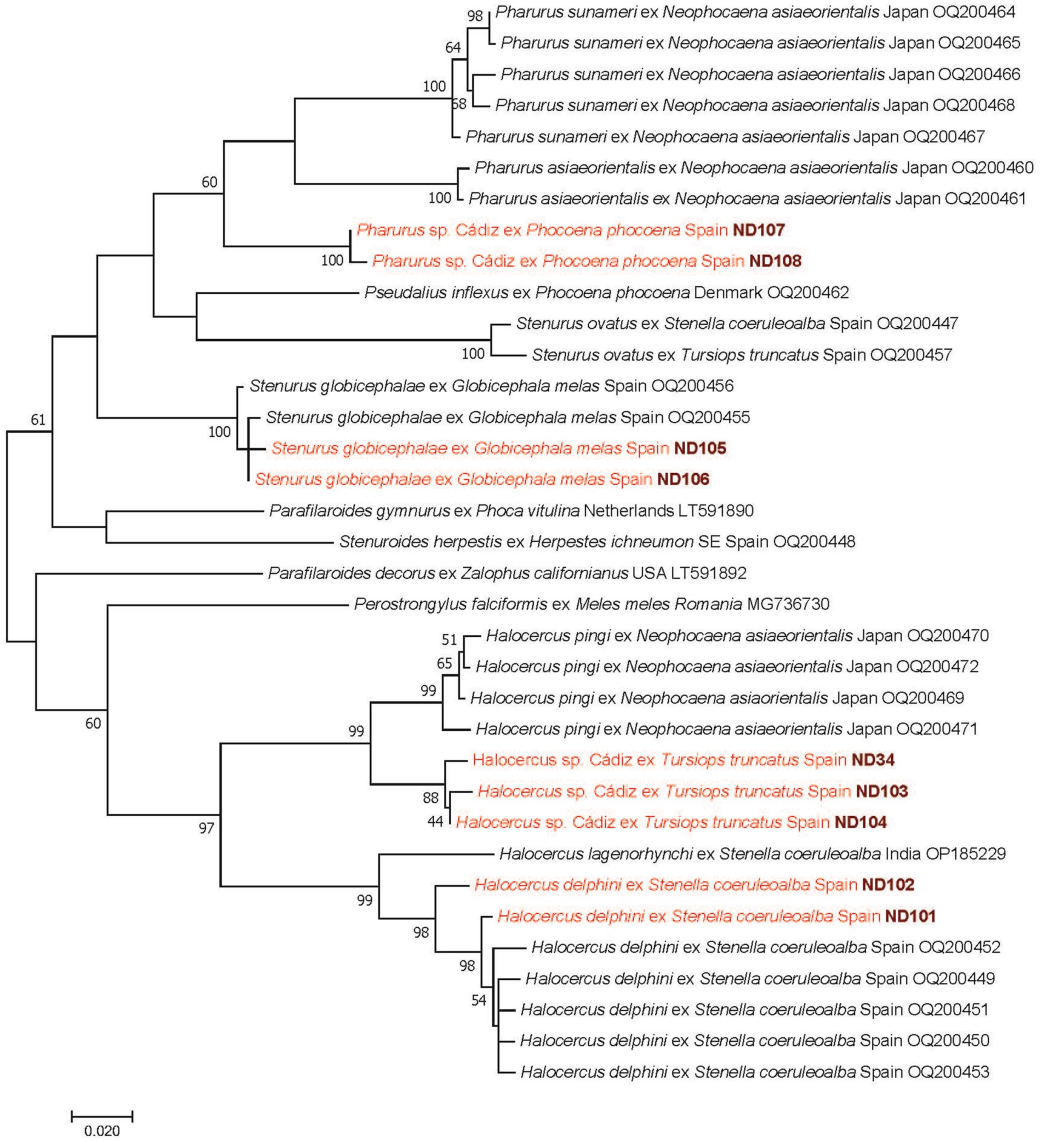
Phylogenetic tree of roundworms (Nematoda: Pseudaliidae) based on the *cox1* gene. In each row of sequences, after the helminth species name, the isolation source (host), the country or region of origin, and the GenBank accession number are shown. Sequences from this study are indicated with red fonts and bold, maroon accession numbers. The evolutionary history was inferred by using the maximum likelihood method based on the Hasegawa–Kishino–Yano model. The tree is drawn to scale, with branch lengths measured in the number of substitutions per site. The analysis involved 35 nucleotide sequences. All positions containing gaps and missing data were eliminated. There were a total of 561 positions in the final dataset.

In the case of the lungworms from *S. coeruleoalba* and *G. melas*, ITS2 sequences confirmed the above species, with 100% identity to *H. delphini* (syn. *Skrjabinalius guevarai*: MN747502) and *S. globicephalae* (FJ787303). Based on the clustering of *Halocercus* sp. Cádiz and *Pharurus* sp. Cádiz sequences in the *cox1* phylogenetic tree, these are species different from those already reported in GenBank, with high (99%) or moderately low (60%) support (Figure 4).

## Discussion

Despite the limited sample size in this study, molecular data were obtained for the first time on several species of endoparasites that infect cetaceans in the Western Mediterranean region. Albeit less important in this context, the negative results of this study will also likely encourage further investigations with a similar aim. For instance, no *Acanthamoeba* DNA was detected in the central nervous system, although (A) these opportunistic parasites occur in the marine environment (12), (B) infection can be acquired from contaminated water, and (C) several members of the order Artiodactyla (which includes infraorder Cetacea) are known to be susceptible to these protozoa (14). Similarly, among cystogenic coccidia (Apicomplexa: Sarcocystidae), no evidence was found for any *Sarcocystis* species in the muscle or nervous system of marine mammals examined in this study, although recently a fatal *Sarcocystis* infection was reported from an Atlantic spotted dolphin (*Stenella frontalis*) (2).

However, the general PCR for cystogenic coccidia revealed the presence of *T. gondii* DNA in multiple organs of *D. delphis*. Considering that all previous studies reporting *Toxoplasma*-infection of this cetacean were based on immunological (serological) methods [in Europe: (15, 16)], to the best of our knowledge, this is the first molecular evidence to confirm *T. gondii* in *D. delphis* in a worldwide context. Disseminated toxoplasmosis was reported to cause stranding among cetaceans, both in Europe (17) and in other parts of the globe (18). In addition, PCR-positivity of the cerebrum and cerebellum also attests that this condition may have played a role in the stranding of the relevant animal, as the latter part of the CNS is particularly important in coordination and maintaining balance (19).

Among marine mammals, cetaceans have a higher prevalence of infection with *T. gondii* in the region of Europe than pinnipeds [14.8% vs. 2.8%: (11)]. For dolphins, the most likely source of acquiring *Toxoplasma* is probably not direct waterborne exposure [as they drink little or no water (6)], but rather via their prey items, most notably fish, which are frequent carriers of this protozoan parasite [prevalence in Europe: 21.8%: (11)]. Importantly, based on the present results, which indicated multi-organ involvement, and according to previous studies reporting high seroprevalence of toxoplasmosis in *D. delphis* (15, 16), this dolphin species should rank among highly *Toxoplasma*-susceptible dolphins, which hitherto included the common bottlenose dolphin, the striped dolphin in the Mediterranean Sea, and many other marine ecosystems worldwide (7).

Among phyllobothriidian cestodes, *C. delphini* and *C. grimaldii* were identified in the blubber of a long-finned pilot whale. These tapeworm species are known to occur in this and other cetacean species in marine habitats surrounding Spain (20), frequently infecting the same host individual simultaneously (21), as also observed here. Both cestode species have a relatively broad host range and may reach high (exceeding 70%) prevalence among dolphins and whales in the Mediterranean and North Atlantic regions (22). In the life cycle of *C. delphini*, cetaceans are the second intermediate host, and sharks play the final host role (23).

This is the first report on the molecular analysis of the ITS2 sequence of *C. delphini* and *C. grimaldii* and of two parts of the *cox1* gene in the case of the former species. As shown here based on sequences of two genetic markers (Figure 2; Supplementary Figure S1), *C. delphini* and *C. grimaldii* are phylogenetically closely related to the member(s) of this genus from pinnipeds (24) and form a monophyletic clade with other *Clistobothrium* species, as already reported based on shorter *28S rRNA* sequences (21), further justifying their taxonomic placement in this genus instead of *Phyllobothrium* (25). Together with other members of the family Phyllobothriidae, *C. delphini* belonged to a sister group of the clade that includes Proteocephalidae, as previously reported based on other nuclear genetic markers (26).

Flukes (Digenea: Brachycladiidae) collected from the peritoneum and liver of a short-beaked common dolphin were molecularly identified as *B. atlanticum*, whereas those removed from the pylorus, gallbladder, and hepatic ducts of a striped dolphin were phylogenetically shown to belong to a different, probably new species (*Brachycladium* sp. Cádiz). The genus *Brachycladium* currently comprises nine species, of which five occur in the Pacific Ocean and five in the Atlantic Ocean (*B. goliath* in both oceans); but only *B. atlanticum* has been reported so far in the Mediterranean Sea (27). So far, only two *Brachycladium* species have been reported in the European region: *B. atlanticum* in both *D. delphis* and *S. coeruleoalba* (28, 29) and *B. goliath* in minke whale (*Balaenoptera acutorostrata*) in the Northeast Atlantic (30). Therefore, the findings of the present study indicate one more species indigenous to the Mediterranean Sea (probably also in marine environments surrounding Europe).

The life cycle of *Brachycladium* spp. is not completely known (30), but most likely fish act as second intermediate hosts, similar to some other members of the Brachycladiidae associated with pinnipeds (31). Although in other, geographically distant marine ecosystems, unidentified *Brachycladium* species were reported (29), to the best of our knowledge, this is the first finding of a third species of this genus in the Mediterranean region. This potentially new species is genetically most closely related to *B. goliath*, in contrast to *B. nipponicum*, which is closer related to *B. atlanticum* (29). Regarding the clinicopathological significance of these species and others with similar tissue tropism, trematodes of dolphins associated with the liver are regarded as potential causes of stranding (32).

Among the lungworms (Nematoda: Pseudaliidae) that were found in four cetacean species in this study, *Halocercus* species were only identified in two species of dolphins (Delphinidae: Delphininae). These mammals play a final host role in the life cycle and probably acquire *Halocercus* infection from fish intermediate hosts (33). However, depending on the species, prenatal infection might also be possible (34).

Regarding the host range of these lungworms, among the Western Mediterranean dolphins analyzed here, *H. delphini* was present in *S. coeruleoalba* and a potentially new species (*Halocercus* sp. Cádiz) in *T. truncatus*. Considering other studies involving the latter dolphin species in Europe, either *H. delphini* (20) or no *Halocercus* sp. were found (22). Since the lungworm species reported here in *T. truncatus* was most closely related to *H. pingi*, it is a species different from the one discovered recently in orca (*Orcinus orca*) in Northern Europe (13).

In a geographical context, three *Halocercus* species were reported to occur along the Spanish Atlantic coastline: *H. delphini* in *D. delphis* and *T. truncatus*, and *H. invaginatus* in *P. phocoena*, as well as an unidentified *Halocercus* sp. in *S. coeruleoalba* (20). More recently, *H. delphini* was shown to have a broader host range along the Mediterranean coastline of Spain, including not only *D. delphis* and *T. truncatus* but also five *Stenella* spp., all from Delphininae (35).

Accepting that the genus *Halocercus* consists of 12 species (36), because *Skrjabinalius cryptocephalus* deserves its own genus and *Skrjabinalius guevarai* is a synonym of *H. delphini* (37), so far there have been five species reported in Europe (38, 39). Since *H. pingi* occurs in the Pacific Ocean (40), this is the first report of a *Halocercus* sp. genetically most closely related to this species in the Western Palearctic.

In the present study, from the pterygoid sinus of *G. melas* (Odontoceti: Delphinidae: Globicephalinae), *Stenurus globicephalae* was identified. This parasite–host association was already reported along the Spanish Atlantic coast (20), and later this pseudaliid worm turned out to have a much broader host range in the Western Mediterranean, including six species of five genera from the subfamily Globicephalinae (35). In addition, in a *P. phocoena* from this study, a *Pharurus* species (tentatively called *Pharurus* sp. Cádiz) was demonstrated, which was molecularly and phylogenetically most closely related to *Phanurus sunameri* [OQ200467: (40)]. Considering that hitherto nematodes of this genus were not known to infect harbor porpoises (41), this is the first report of a lungworm species from this cetacean that, based on two genetic markers, aligns with *Phanurus* species and belongs to their phylogenetic group.

Taken together, the phylogenetic relationships of the four lungworm species found in this study confirm (A) previously reported relative host-specificity of pseudaliid worms, particularly among Delphinidae (35), and (B) that *Halocercus* vs. *Stenurus* and *Pharurus* species belong to two sister groups (40). In addition, (C) clustering of these genera also attests that the family Pseudaliidae is not monophyletic unless including *Parafilaroides* and *Perostrongylus*, as proposed earlier (40). Importantly, pseudaliid lungworms of cetaceans (especially if they occur in cranial sinuses) are thought to play a role in the stranding of infected animals (42).

## Conclusion

In this study, first-time molecular evidence was obtained on the organ-specific location of *T. gondii* in *D. delphis*. Molecular-phylogenetic analyses of this study revealed an unexpected diversity of helminth parasites in cetaceans of the Western Mediterranean, including hitherto not-yet-analyzed genetic markers of long-known species (as exemplified by the cestode *C. delphini*) and possibly even undescribed (new) species (among flukes and lungworms in the genera *Brachycladium* and *Halocercus*, respectively). These results will substantially contribute to understanding host–parasite interactions and eco-epidemiological risks that might threaten cetacean populations in the Mediterranean Sea or Northeast Atlantic region.

## Data Availability

The sequences obtained during this study are deposited in GenBank under the accession numbers listed in Table 1. All other relevant data are included in the manuscript and the references or are available upon request by the corresponding author.

## Footnotes

^1^ https://blast.ncbi.nlm.nih.gov

## References

[1] Fordyce RE. Cetacea (whales, porpoises and dolphins) In: Encyclopedia of life sciences. 1st ed. ed. Ewan Fordyce R.. Chichester: John Wiley & Sons, Ltd, (2001).

[2] Balik SE Ossiboff RJ Stacy NI Wellehan JF Huguet EE Gallastegui A. Case report: Sarcocystis speeri, aspergillus fumigatus, and novel Treponema sp. infections in an adult Atlantic spotted dolphin (Stenella frontalis). Front Vet Sci. (2023) 10:1132161. doi: 10.3389/fvets.2023.1132161, PMID: 37077953 PMC10106728

[3] Guégan JF. Chlimate change and infectious diseases in the Mediterranean region In: The Mediterranean region under climate change: A scientific update [internet]. eds. Marie-LiseSabrié Gibert-Brunet E Mourier T Mareille: IRD Éditions, (2016).

[4] Dupke S Buchholz U Fastner J Förster C Frank C Lewin A. Impact of climate change on waterborne infections and intoxications. J Health Monit. (2023) 8:62. doi: 10.25646/11402 37342430 PMC10278370

[5] Cuvertoret-Sanz M López-Figueroa C Byrne AO Canturri A Martí-Garcia B Pintado E. Causes of cetacean stranding and death on the Catalonian coast (western Mediterranean Sea), 2012-2019. Dis Aquat Org. (2020) 142:239–53. doi: 10.3354/dao03550, PMID: 33331291

[6] Dubey JP Zarnke R Thomas NJ Wong SK Van Bonn W Briggs M. Toxoplasma gondii, Neospora caninum, Sarcocystis neurona, and Sarcocystis canis-like infections in marine mammals. Vet Parasitol. (2003) 116:275–96. doi: 10.1016/S0304-4017(03)00263-2, PMID: 14580799

[7] Di Guardo G Mazzariol S. Toxoplasma gondii: clues from stranded dolphins. Vet Pathol. (2013) 50:737. doi: 10.1177/0300985813486816 24014612

[8] Gardner BR Bachmann NL Polkinghorne A Hufschmid J Tadepalli M Marenda M. A novel marine mammal Coxiella burnetii—genome sequencing identifies a new genotype with potential virulence. Pathogens. (2023) 12:893. doi: 10.3390/pathogens12070893 37513739 PMC10386718

[9] Marangi M Carlucci R Carlino P Fanizza C Cirelli G Maglietta R. Dolphins and sea turtles may host zoonotic parasites and pathogenic bacteria as indicators of anthropic pressure in the Gulf of Taranto (northern Ionian Sea, central-eastern Mediterranean Sea). Vet Res Commun. (2022) 46:1157–66. doi: 10.1007/s11259-022-10011-y 36190602 PMC9684234

[10] Reeves RR Notarbartolo di Sciara G. The status and distribution of cetaceans in the Black Sea and Mediterranean Sea. (2006) [cited 2023. december 18.]; Available at: https://policycommons.net/artifacts/1376036/the-status-and-distribution-of-cetaceans-in-the-black-sea-and-mediterranean-sea/1990298/

[11] Ahmadpour E Rahimi MT Ghojoghi A Rezaei F Hatam-Nahavandi K Oliveira SMR. Toxoplasma gondii infection in marine animal species, as a potential source of food contamination: a systematic review and Meta-analysis. Acta Parasitol. (2022) 67:592–605. doi: 10.1007/s11686-021-00507-z, PMID: 35038109 PMC8761968

[12] Mohd Hussain RH Abdul Ghani MK Khan NA Siddiqui R Anuar TS. Acanthamoeba species isolated from marine water in Malaysia exhibit distinct genotypes and variable physiological properties. J Water Health. (2022) 20:54–67. doi: 10.2166/wh.2021.128, PMID: 35100154

[13] Lehnert K Boyi JO Siebert U. Potential new species of pseudaliid lung nematode (Metastrongyloidea) from two stranded neonatal orcas (Orcinus orca) characterized by ITS -2 and COI sequences. Ecol Evol. (2023) 13:e10036. doi: 10.1002/ece3.10036 37139403 PMC10150029

[14] Schuster FL Visvesvara GS. Free-living amoebae as opportunistic and non-opportunistic pathogens of humans and animals. Int J Parasitol. (2004) 34:1001–27. doi: 10.1016/j.ijpara.2004.06.004, PMID: 15313128

[15] Cabezón O Resendes AR Domingo M Raga JA Agustí C Alegre F. Seroprevalence of toxoplasma gondii antibodies in wild dolphins from the Spanish Mediterranean coast. J Parasitol. (2004) 90:643–4. doi: 10.1645/GE-257R, PMID: 15270114

[16] Forman D West N Francis J Guy E. The sero-prevalence of toxoplasma gondii in British marine mammals. Mem Inst Oswaldo Cruz. (2009) 104:296–8. doi: 10.1590/S0074-02762009000200024, PMID: 19430656

[17] Fernández-Escobar M Giorda F Mattioda V Audino T Di Nocera F Lucifora G. Toxoplasma gondii genetic diversity in Mediterranean dolphins. Pathogens. (2022) 11:909. doi: 10.3390/pathogens11080909 36015030 PMC9416038

[18] Landrau-Giovannetti N Waltzek TB López-Orozco N Su C Rotstein D Levine G. Prevalence and genotype of toxoplasma gondii in stranded Hawaiian cetaceans. Dis Aquat Org. (2022) 152:27–36. doi: 10.3354/dao03699, PMID: 36394138

[19] Kern A Seidel K Oelschläger HHA. The central vestibular complex in dolphins and humans: functional implications of Deiters’ nucleus. Brain Behav Evol. (2009) 73:102–10. doi: 10.1159/000213646, PMID: 19390175

[20] Abollo E Lopez A Gestal C Benavente P Pascual S. Macroparasites in cetaceans stranded on the northwestern Spanish Atlantic coast. Dis Aquat Org. (1998) 32:227–31. doi: 10.3354/dao032227, PMID: 9676247

[21] Agusti C Aznar FJ Olson PD Littlewood DTJ Kostadinova A Raga JA. Morphological and molecular characterization of tetraphyllidean merocercoids (Platyhelminthes: Cestoda) of striped dolphins (Stenella coeruleoalba) from the Western Mediterranean. Parasitology. (2005) 130:461–74. doi: 10.1017/S0031182004006754, PMID: 15830821

[22] Terracciano G Fichi G Comentale A Ricci E Mancusi C Perrucci S. Dolphins stranded along the tuscan coastline (Central Italy) of the “pelagos sanctuary”: a parasitological investigation. Pathogens. (2020) 9:612. doi: 10.3390/pathogens9080612, PMID: 32727040 PMC7459703

[23] Aznar FJ Agustí C Littlewood DTJ Raga JA Olson PD. Insight into the role of cetaceans in the life cycle of the tetraphyllideans (Platyhelminthes: Cestoda). Int J Parasitol. (2007) 37:243–55. doi: 10.1016/j.ijpara.2006.10.010, PMID: 17161403

[24] Klotz D Hirzmann J Bauer C Schöne J Iseringhausen M Wohlsein P. Subcutaneous merocercoids of Clistobothrium sp. in two cape fur seals (Arctocephalus pusillus pusillus). Int J Parasitol Parasites Wild. (2018) 7:99–105. doi: 10.1016/j.ijppaw.2018.02.003, PMID: 29988787 PMC6032031

[25] Caira JN Jensen K Pickering M Ruhnke TR Gallagher KA. Intrigue surrounding the life-cycles of species of Clistobothrium (Cestoda: Phyllobothriidea) parasitising large pelagic sharks. Int J Parasitol. (2020) 50:1043–55. doi: 10.1016/j.ijpara.2020.08.002, PMID: 32979336

[26] Caira JN Jensen K Waeschenbach A Olson PD Littlewood DTJ. Orders out of chaos–molecular phylogenetics reveals the complexity of shark and stingray tapeworm relationships. Int J Parasitol. (2014) 44:55–73. doi: 10.1016/j.ijpara.2013.10.004, PMID: 24275646 PMC4834996

[27] Fraija-Fernández N Aznar F Raga J Gibson D Fernández M. A new brachycladiid species (Digenea) from Gervais’ beaked whale Mesoplodon europaeus in North-Western Atlantic waters. Acta Parasitol [Internet]. (2014) 59:510–7. doi: 10.2478/s11686-014-0274-7, PMID: 25119367

[28] Mateu P Raga JA Aznar FJ. Host specificity of Oschmarinella rochebruni and Brachycladium atlanticum (Digenea: Brachycladiidae) in five cetacean species from western Mediterranean waters. J Helminthol. (2011) 85:12–9. doi: 10.1017/S0022149X10000180, PMID: 20359374

[29] Kim S Youn H Lee K Lee H Kim MJ Kang Y. Novel morphological and molecular data for Nasitrema spp.(Digenea: Brachycladiidae) in the east Asian finless porpoise (Neophocaena asiaeorientalis sunameri). Front Mar Sci. (2023) 10:1187451. doi: 10.3389/fmars.2023.1187451

[30] Briscoe AG Bray RA Brabec J Littlewood DTJ. The mitochondrial genome and ribosomal operon of Brachycladium goliath (Digenea: Brachycladiidae) recovered from a stranded minke whale. Parasitol Int. (2016) 65:271–5. doi: 10.1016/j.parint.2016.02.004, PMID: 26883466

[31] Kremnev G Gonchar A Krapivin V Knyazeva O Krupenko D. First elucidation of the life cycle in the family Brachycladiidae (Digenea), parasites of marine mammals. Int J Parasitol. (2020) 50:997–1009. doi: 10.1016/j.ijpara.2020.05.011, PMID: 32663502

[32] Ridgway SH Dailey MD. Cerebral and cerebellar involvement of trematode parasites in dolphins and their possible role in stranding. J Wildl Dis. (1972) 8:33–43. doi: 10.7589/0090-3558-8.1.33, PMID: 5007899

[33] Lehnert K von Samson-Himmelstjerna G Schaudien D Bleidorn C Wohlsein P Siebert U. Transmission of lungworms of harbour porpoises and harbour seals: molecular tools determine potential vertebrate intermediate hosts. Int J Parasitol. (2010) 40:845–53. doi: 10.1016/j.ijpara.2009.12.008, PMID: 20123100

[34] Dailey M Walsh M Odell D Campbell T. Evidence of prenatal infection in the bottlenose dolphin (Tursiops truncates) with the lungworm Halocercus lagenorhynchi (Nematoda: Pseudaliidae). J Wildl Dis. (1991) 27:164–5. doi: 10.7589/0090-3558-27.1.164 2023318

[35] Pool R Romero-Rubira C Raga JA Fernández M Aznar FJ. Determinants of lungworm specificity in five cetacean species in the western Mediterranean. Parasit Vectors. (2021) 14:április 12:196. doi: 10.1186/s13071-021-04629-1 PMC8042974 33845871

[36] de Oliveira Carvalho Demarque I FCR d O da Silveira LS Barbosa LA Ederli NB. The lungworm, Halocercus brasiliensis (Nematoda: Pseudaliidae), from Guiana dolphins Sotalia guianensis from Brazil with pathological findings. J Parasitol. (2020) 106:254–60. doi: 10.1645/19-77 32206795

[37] Pool R Fernández M Chandradeva N Raga JA Aznar FJ. The taxonomic status of Skrjabinalius guevarai Gallego & Selva, 1979 (Nematoda: Pseudaliidae) and the synonymy of Skrjabinalius Delyamure, 1942 and Halocercus Baylis & Daubney, 1925. Syst Parasitol. (2020) 97:389–401. doi: 10.1007/s11230-020-09921-9 32533535

[38] Gibson DI Harris EA Bray RA Jepson PD Kuiken T Baker JR. A survey of the helminth parasites of cetaceans stranded on the coast of England and Wales during the period 1990-1994. J Zool. (1998) 244:563–74. doi: 10.1111/j.1469-7998.1998.tb00061.x

[39] Costello M Emblow C White R. European register of marine species: A check-list of the marine species in Europe and a bibliography of guides to their identification In: Collection Patrimoines Naturels. eds. Costello MJ Emblow C White R Paris: Muséum National d’Histoire Naturelle, Institute d’écologie et de gestion de la biodiversité service du patrimoine naturel. (2001). 463.

[40] Pool R Shiozaki A Raga JA Fernández M Aznar FJ. Molecular phylogeny of the Pseudaliidae (Nematoda) and the origin of associations between lungworms and marine mammals. Int J Parasitol Parasites Wildl. (2023) 20:192–202. doi: 10.1016/j.ijppaw.2023.03.002, PMID: 36969083 PMC10034209

[41] Dzido J Rolbiecki L Izdebska JN Rokicki J Kuczkowski T Pawliczka I. A global checklist of the parasites of the harbor porpoise Phocoena phocoena, a critically-endangered species, including new findings from the Baltic Sea. Int J Parasitol Parasites Wildl. (2021) 15:290–302. doi: 10.1016/j.ijppaw.2021.07.002, PMID: 34336594 PMC8313437

[42] Fischbach JR Seguel M. A systematic review of the diversity and virulence correlates of metastrongyle lungworms in marine mammals. Parasitology. (2023) 150:1–44. doi: 10.1017/S0031182023001014 PMC10801380 37859401

