# Supplementary material for: Molecular investigation of endoparasites of marine mammals (Cetacea: Mysticeti, Odontoceti) in the Western Mediterranean
Source: Front Vet Sci. 2024 Sep 10;11:1431625. doi: 10.3389/fvets.2024.1431625 (PMC11420046; doi:10.3389/fvets.2024.1431625)
Supplement: Supplementary file 1 [file Data_Sheet_1.PDF]

Supplementary Table 1. Samples used in this study, collection data and results of molecular analyses.

**Parvorder:** Family	**Species**	**Collection data**		**PCR-tested organs**								**PCR-tested parasite group [number of specimens analysed]**
		**Date (day-month-year)**	**Location: province (city if known)**	**Muscle**	**Lungs**	**Liver**	**Spleen**	**Blood**	**Medulla**	**Cerebellum**	**Encephalon**	
**Odontoceti**: Delphinidae	Short-beaked common dolphin (*Delphinus delphis*)	12-08-2022	Cádiz	1	1	1	1	1	-	1	1	-
	Short-beaked common dolphin (*Delphinus delphis*)	26-11-2022	Málaga (Nerja)	-	-	1	-	-	-	-	-	Flukes (*Brachycladium*) [1]
	Short-beaked common dolphin (*Delphinus delphis*)	27-12-2022	Granada (Granada)	-	1	1			1	1	1	-
	Striped dolphin (*Stenella coeruleoalba*)	15-08-2022	Cádiz	1	1	1	1	1		1	1	-
	Striped dolphin (*Stenella coeruleoalba*)	15-11-2022	Cádiz (La Línea de la Concepción)	-	1	1	-	-	-	-	-	Lungworms (*Halocercus*) [2]<br>Flukes (*Brachycladium*) [3]
	Common bottlenose dolphin (*Turisops truncatus)*	13-12-2022	Cádiz (Chipiona)	1		1	-	-	-	-	-	Lungworms (*Halocercus*) [3]
	Long-finned pilot whale (*Globicephala melas*)	19-12-2022	Málaga	-	-	-	-	-	-	-	-	Lungworms (*Stenurus*)<br>Cestodes (*Clistobothrium*) [2]
**Odontoceti**: Phocoenidae	Harbor porpoise (*Phoconea phoconea*)	23-08-2022	Huelva	1	1	1	1	-	-	-	-	Lungworms (*Pharurus*)
**Mysticeti**: Balaenopteridae	Fin whale (*Balaenoptera physalus)*	23-12-2022	Cádiz (Bay of Algeciras)	1	1	-	-	1	-	-	-	-
