# Supplementary material for: Molecular investigation of endoparasites of marine mammals (Cetacea: Mysticeti, Odontoceti) in the Western Mediterranean
Source: Front Vet Sci. 2024 Sep 10;11:1431625. doi: 10.3389/fvets.2024.1431625 (PMC11420046; doi:10.3389/fvets.2024.1431625)
Supplement: Supplementary file 2 [file Data_Sheet_2.pdf]

**Supplementary Table 2:** Primers and details for conventional PCR methods used in this study.

| | Target group | Target gene | Primer name | Primer sequence (5'-3') | Amplicon length (bp) | Thermocycling profile | Reference |
|---|---|---|---|---|---|---|---|
| Protozoa | Cystogenic coccidia | SSU | COC1*<br>COC2* | AAG TAT AAG CTT TTA TAC GGC T<br>CAC TGC CAC GGT AGT CCA ATA C | ~350 | 95 °C for 10 min; 40× (94 °C for 30 s; 54 °C for 30 s; 72 °C for 30 s); 72 °C for 10 min | Ho et al., 1996 |
| | *Toxoplasma gondii* | repeat region | TOX-8 (fw)<br>TOX5 (rev) | CCC AGC TGC GTC TGT CGG GAT<br>CGC TGC AGA CAC AGT GCA TCT GGA TT | ~480 | 95 °C for 5 min; 35× (95 °C for 40 s; 60 °C for 1 min; 72 °C for 1 min); 72 °C for 10 min | Homan et al., 2000; Reischl et al., 2003; Schares et al., 2008 |
| | *Acanthamoeba* spp. | 18S rRNA | JDP1<br>JDP2 | GGC CCA GAT CGT TTA CCG TGA A<br>TCT CAC AAG CTG CTA GGG GAG TCA | ~480 | 95 °C for 5 min; 35× (95 °C for 35 s; 56 °C for 45 s; 72 °C for 1 min); 72 °C for 7 min | Niyyati et al., 2016 |
| Helminths | Nematoda (general barcode) | cox1 | LCO1490<br>HCO2198 | GGT CAA CAA ATC ATA AAG ATA TTG G<br>TAA ACT TCA GGG TGA CCA AAA AAT CA | ~710 | 95 °C for 5 min; 40× (94 °C for 40 s; 48 °C for 1 min; 72 °C for 1 min); 72 °C for 10 min | Folmer et al., 1994 |
| | Nematoda (Pseudaliidae) | ITS2 | PseuITS2F<br>PseuITS2R<br><br>LWf<br>LWr | CCT TCG GCA CAT CTT GTT CA<br>GGG TAA TCA CAT CTG AGT TCA<br><br>GCA GAC GCT TAG AGT GGT GAA A<br>ACT CGC CGT TAC TAA GGG AAT C | ~550<br><br>~650 | 95 °C for 5 min; 40× (95 °C for 30 s; 59 °C for 30 s; 72 °C for 50 s); 72 °C for 10 min | Pool et al., 2023<br><br>Lehnert et al., 2023 |
| | Trematoda, Cestoda | cox1 | JB3<br>JB4.5 | TTT TTT GGG CAT CCT GAG GTT TAT<br>TAA AGA AAG AAC ATA ATG AAA ATG | ~450 | 95 °C for 5 min; 40× (95 °C for 40 s; 55 °C for 1 min; 72 °C for 1,5 min); 72 °C for 10 min | Bowles et al., 1993; Wang et al., 2012 |
| | Trematoda, Cestoda | cox1 | Dice1F<br>Dice11R | ATT AAC CCT CAC TAA ATT WCN TTR GAT CAT AAG<br>TAA TAC GAC TCA CTA TAG CWG WAC HAA ATT THC GAT C | ~680 | 94 °C for 5 min; 3× (94 °C for 40 s, 51 °C for 40 s, 72 °C for 1 min); 5× 'touchdown' (94 °C for 40 s, 50 °C to 46 °C for 40 s (dropping 1 °C per cycle), 72 °C for 1 min); 35× (94 °C for 40 s, 45 °C for 40 s, 72 °C for 1 min); 72 °C for 5 min | Van Steenkiste et al., 2016 |
| | Trematoda, Cestoda | ITS2 | 3S-fw<br>A28S-rev | GGT ACC GGT GGA TCA CTC GGC TCG TG<br>GGG ATC CTG GTT AGT TTC TTT TCC TCC GC | ~522 | 95 °C for 5 min; 40× (95 °C for 40 s; 55 °C for 1 min; 72 °C for 1,5 min); 72 °C for 10 min | Prasad et al., 2007; Sahu et al., 2016 |
| | Cestoda | 18S rRNA | LSU5<br>1200R | TAG GTC GAC CCG CTG AAY TTA AGC<br>GCA TAG TTC ACC ATC TTT CGG | ~1400 | 95 °C for 5 min; 40× (95 °C for 30 s; 54 °C for 40s; 72 °C for 1,5 min); 72 °C for 5 min | Littlewood et al., 2001 |
| | Cestoda | 18S rRNA | 300F<br>ECD2 | CAA GTA CCG TGA GGG AAA GTT<br>CTT GGT CCG TGT TTC AAG ACG GG | ~750 | 95 °C for 5 min; 40× (95 °C for 30 s; 54 °C for 40s; 72 °C for 1,5 min); 72 °C for 5 min | Littlewood et al., 2001 |

Bowles, J., Hope, M., Tiu, W.U., Liu, X., McManus, D.P., 1993. Nuclear and mitochondrial genetic markers highly conserved between Chinese and Philippine *Schistosoma japonicum*. Acta Tropica 55, 217–229. https://doi.org/10.1016/0001-706X(93)90079-Q

Folmer O., Black M., Hoeh W., Lutz R., Vrijenhoek R., 1994. DNA primers for amplification of mitochondrial cytochrome c oxidase subunit I from diverse metazoan invertebrates. Mol. Mar. Biol. Biotechnol., 3, pp. 294-299

Ho, M., Barr, B., Marsh, A., Anderson, M., Rowe, J., Tarantal, A., Hendrickx, A., Sverlow, K., Dubey, J., Conrad, P., 1996. Identification of bovine *Neospora* parasites by PCR amplification and specific small-subunit rRNA sequence probe hybridization. Journal of clinical microbiology 34, 1203–8. https://doi.org/10.1128/JCM.34.5.1203-1208.1996

Homan, W.L., Vercammen, M., De Braekeleer, J., Verschueren, H., 2000. Identification of a 200- to 300-fold repetitive 529 bp DNA fragment in *Toxoplasma gondii*, and its use for diagnostic and quantitative PCR. Int J Parasitol 30, 69–75. https://doi.org/10.1016/s0020-7519(99)00170-8

Lehnert, K., Boyi, J.O., Siebert, U. Potential new species of pseudaliid lung nematode (Metastrongyloidea) from two stranded neonatal orcas (*Orcinus orca*) characterized by ITS-2 and COI sequences. Ecology and Evolution. 2023;13:e10036. https://doi.org/10.1002/ece3.10036

Littlewood DT, Curini-Galletti M, Herniou EA. The interrelationships of proseriata (Platyhelminthes: seriata) tested with molecules and morphology. Mol Phylogenet Evol. 2000 Sep;16(3):449-66. doi: 10.1006/mpev.2000.0802.

Niyyati M, Saberi R, Latifi A, Lasjerdi Z., 2016. Distribution of Acanthamoeba Genotypes Isolated from Recreational and Therapeutic Geothermal Water Sources in Southwestern Iran. Environ Health Insights. 2016 Apr 19;10:69-74. doi: 10.4137/EHI.S38349. PMID: 27127409; PMCID: PMC4838054.

Pool, R., Shiozaki, A., Raga, J.A., Fernández, M., Aznar, J.F. Molecular phylogeny of the Pseudaliidae (Nematoda) and the origin of associations between lungworms and marine mammals. International Journal for Parasitology: Parasites and Wildlife 20 (2023) 192–202. https://doi.org/10.1016/j.ijppaw.2023.03.002

Prasad, P.K., Tandon, V., Chatterjee, A., Bandyopadhyay, S., 2007. PCR-based determination of internal transcribed spacer (ITS) regions of ribosomal DNA of giant intestinal fluke, *Fasciolopsis buski* (Lankester, 1857) Looss, 1899. Parasitol Res 101, 1581–1587. https://doi.org/10.1007/s00436-007-0680-y

Reischl, U., Bretagne, S., Krüger, D., Ernault, P., Costa, J.-M., 2003. Comparison of two DNA targets for the diagnosis of toxoplasmosis by real-time PCR using fluorescence resonance energy transfer hybridization probes. BMC Infect Dis 3, 7. https://doi.org/10.1186/1471-2334-3-7

Sahu, R., Biswal, D.K., Roy, B., Tandon, V., 2016. Molecular characterization of *Opisthorchis noverca* (Digenea: Opisthorchiidae) based on nuclear ribosomal ITS2 and mitochondrial COI genes. J Helminthol 90, 607–614. https://doi.org/10.1017/S0022149X15000851

Schares, G., Herrmann, D.C., Beckert, A., Schares, S., Hosseininejad, M., Pantchev, N., Globokar Vrhovec, M., Conraths, F.J., 2008. Characterization of a repetitive DNA fragment in *Hammondia hammondi* and its utility for the specific differentiation of *H. hammondi* from *Toxoplasma gondii* by PCR. Mol Cell Probes 22, 244–251. https://doi.org/10.1016/j.mcp.2008.04.003

Van Steenkiste N., Locke S.A., Castelin M., Marcogliese D.J., Abbott C.L., 2016. New primers for DNA barcoding of digeneans and cestodes (Platyhelminthes). Mol Ecol Resour. 2015 Jul;15(4):945-52. doi: 10.1111/1755-0998.12358.Niyyati M, Saberi R, Latifi A, Lasjerdi Z., 2016. Distribution of *Acanthamoeba* genotypes isolated from recreational and therapeutic geothermal water sources in Southwestern Iran. Environ Health Insights. 10, 69–74. https://doi: 10.4137/EHI.S38349

Wang, X.-Y., Zhao, G.-H., Liu, G.-H., Li, J.-Y., Zhou, D.-H., Xu, M.-J., Lin, Q., Zhu, X.-Q., 2013. Characterization of *Dicrocoelium chinensis* from domestic yaks in Gansu and Sichuan provinces, China, using genetic markers in two mitochondrial genes. Mitochondrial DNA 24, 263–266. https://doi.org/10.3109/19401736.2012.744974
