# Supplementary figures and images for: Molecular investigation of endoparasites of marine mammals (Cetacea: Mysticeti, Odontoceti) in the Western Mediterranean
Source: Front Vet Sci. 2024 Sep 10;11:1431625. doi: 10.3389/fvets.2024.1431625 (PMC11420046; doi:10.3389/fvets.2024.1431625)

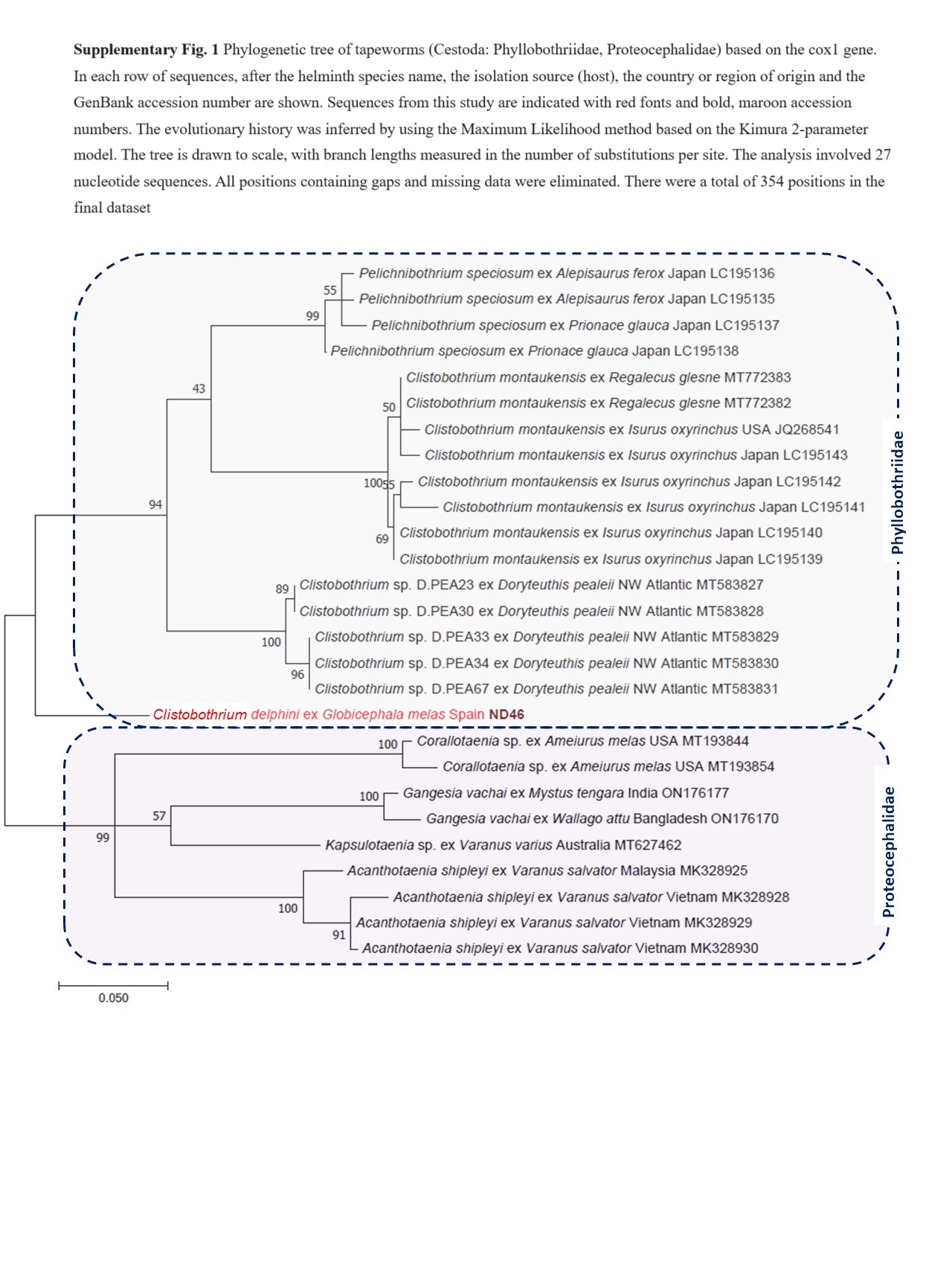

Supplement: Supplementary file 3 [file Image_1.JPEG]
